# Central Park

**Published:** 1860-07

**Authors:** 


" Apothecaries" will go to pot, because there will be no use for their "villainous compounds." The fresh out-door air amid sunshine and fashion and youth and beauty, is more infallibly exhilarating than any brandied " tonic" ever devised—the best anodyne in the world is out-door exercise. No sudorific ever compounded, concocted, macerated or mixed, is more certain of its desired effect, than a walk, or drive, or game in the open air. Nothing so infallibly acts " like a charm" in loosening cough and dislodging phlegm, as wisely conducted out-door activities. Let the experience of any man give its certificate, signed, sealed, acknowledged and sworn to, of the appetizing effects of five miles on horseback "and repeat." Does any body, can any body have the impudence to assert that there is any better " Liver pill" in the world than a two-hours' trot in the saddle at a two-forty pace? The most unsophisticated ninny of a "first course" student knows that if the appetite is right, the skin perspires, the cough is loose, and the " liver works," a man can't die of any thing except of love or a bullet, or some other incongruous concatenation of circumstances. The Central Park then being a panacea for all sickness, drugs and druggery must go by the board, swilling liquor under the guise of "tonics," will be done away with, which by the way has unsuspectingly lured many a man to the bottle, and many a splendid woman to the poppy, to fade away from the society they once ornamented to go down eventually to a contemptuous grave; so that the great Central Park of New-York City, with its eight hundred and forty-four acres of hill and dale, of field and woodland, of rock and plain, will not only be a splendid gymnasium, but a free dispensatory, not of drugs! but of health, and a grand Temple of Temperance.

But there is one point which yet remains to be clarified. How, under these circumstances is New-York to become the paradise of doctors, the regulars we mean! for we throw overboard as unfit for ballast even, the steamer, the mesmerist, the clairvoyant, the cold waterist and the infinitesimal, *et id omne* class of vagarists, for none of them ever had an idea which was not derived from the investigations, researches, and studies of recognized medicine; nor can the whole cavalcade of them name a single authenticated discovery in anatomy, physiology, surgery, or physic, nor indeed in any thing else, unless in certain forms of vulgar legerdemain, by which a transference of "meum and teum" is effected by means of oil or fog, without any honorable "quid pro quo." But let that go down into the tomb of all the Capulets.

Scientific medicine will flourish in the dawning Gothamite millennium in this wise. Its legitimate object is to teach the people how to keep well, how to avoid disease, the high and first "chair" in which "school" was conceived, founded, inaugurated and occupied by the editor of the JOURNAL OF HEALTH. But there will be cases, not a few, where there will be causes of mishap and disease which no human sagacity can either foresee or prevent; in such cases, the educated practitioner will be called in to ascertain the exact "state of the case," and to "prescribe," not drugs, but such forms of activities and such rules of dress, rest, eating, drinking and sleeping, as will most happily meet the requirements of each particular patient; then by degrees, curing disease without physic, even in homeopathic quantities, will become more and more a science, educated allopathy will be king, and New-York become the home and the Temple of Hygeia!

Nor is this day indefinitely distant. Says the *Daily Times*, already are the available spots of the Central Park visited by large numbers on fine days; citizens of all classes, from the millionaire to the humble artisan, may be seen enjoying the delightful walks and drives, and snuffing the invigorating atmosphere. When the boats and cheap hacks and the grounds for the ball-players, cricketers, and military are finished, the Park will present on fine days, a scene of brilliancy and picturesqueness unequaled by a Venetian carnival; and that day! thank the energy and fidelity of the Commissioners, is not far distant. Already has it become a fashionable resort. On any fine day at five in the afternoon may be seen a display of equipages quite surpassing any thing of the kind on this continent. Horseback-riding is coming more into vogue since a place is provided where that health and grace-provoking amusement can be comfortably prosecuted. Not a few families have changed their hour of dining in order to be able to ride or drive at a time when the world is to be seen. Not a few persons of means, who heretofore have not cared to keep carriages and horses merely to drive around town, have obtained them recently. Livery-stable keepers have not open vehicles enough to supply the demand of those who wish to hire. The Park is all the rage; it is more frequented and more talked about than the opera; the imported swans are discussed more vigorously than the prima-donnas; riding-habits are in greater demand than opera-cloaks, and a new bridge on a new road provokes more comment even than the *débût* of another tenor, or the announcement of another work of Verdi. The leaders of the ton have agreed to make Wednesday and Saturday the fashionable days for the drive. Reader, let us be there to see!

English tourists may remember that the Oxford students have boats for rowing, sharp, narrow and long, holding but one person only, and in these they spend many an hour, acquiring by degrees an agility of management and a rapidity of motion very remarkable. It is greatly to be desired that some of these should be promptly prepared for use on the lake in the Central Park under such regulations as will best secure their proper and general use. There is perhaps no form of mere exercise so well calculated to bring into free play the whole muscular system, and so specially well calculated to develop the chest and the strength of the arms; its advantages to girls would be almost incalculable.

